# Expression and functions of galectin-7 in ovarian cancer

**DOI:** 10.18632/oncotarget.2299

**Published:** 2014-07-31

**Authors:** Marilyne Labrie, Maria Claudia Vladoiu, Andrée-Anne Grosset, Louis Gaboury, Yves St-Pierre

**Affiliations:** ^1^ INRS-Institut Armand-Frappier, Laval, Québec, Canada; ^2^ Institute for Research in Immunology and Cancer, Downtown Station, Montréal, Québec, Canada

**Keywords:** galectin-7, ovarian cancer, immunosupression, p53, MMP-9

## Abstract

There is a critical need to develop effective new strategies for diagnosis and treatment of ovarian cancer. In the present work, we investigated the expression of galectin-7 (gal-7) in epithelial ovarian cancer (EOC) cells and studied its functional relevance. Immunohistochemical analysis of gal-7 expression in tissue microarrays showed that while gal-7 was not detected in normal ovarian tissues, positive cytoplasmic staining of gal-7 was detected in epithelial cells in all EOC histological subtypes but was more frequent in high grade tumors and metastatic samples. Gal-7 expression correlated with a significant difference in the overall survival of patients with ovarian serous cystadenocarcinoma. Furthermore, using human EOC cell lines, we found that gal-7 expression was induced by mutant p53. Mechanistically, Matrigel invasion assays and live cell imaging showed that gal-7 increased the invasive behavior of ovarian cancer cells by inducing MMP-9 and increasing cell motility. EOC cells can also secrete gal-7. Recombinant human gal-7 kills Jurkat T cells and human peripheral T cells, suggesting that gal-7 also has immunosuppressive properties. Taken together, our study validates the clinical significance of gal-7 overexpression in ovarian cancer and provides a rationale for targeting gal-7 to improve the outcome of patients with this disease.

## INTRODUCTION

Epithelial ovarian cancer (EOC) accounts for approximately 85–90% of total ovarian cancers and is the leading cause of gynecologic malignancies in North America and worldwide [1]. Despite improvements in various treatments, the 5-year survival of patients with advanced stage disease remains less than 40%. Largely, this is due to the fact that EOC tends to be diagnosed at advanced stages, the lack of effective treatments for patients and incomplete understanding of the molecular underpinnings of the disease progression. Therefore, it is essential to identify potential biomarkers and novel therapeutic targets that are involved in ovarian cancer initiation, progression and outcome to help improve clinical outcomes.

Members of the galectin family are recognized for their ability to bind β-galactosides via their carbohydrate recognition domain (CRD). They play an important role in several physiological processes including cell migration, programmed cell death and regulation of the immune response (as reviewed in [2]). They are also involved in a number of pathological conditions including cancer. In fact, galectins have been shown to induce systemic and local immunosuppression by killing immune cells. They can also bind to the surface of tumor cells to form cell surface lattices that stabilize expression of key growth factor receptors. In doing this, galectins increase cell growth, motility, and the overall invasive properties of cancer cells [3, 4]. However, so far most of the attention has been focused on galectin-1 and galectin-3, and we still know very little about how gal-7 expression affects cancer progression and its distinctive role. Unlike other galectins, gal-7 is strictly expressed in epithelial cells. In fact, this galectin is generally considered to be a marker of epithelial differentiation. It is present in normal epithelial cells of various tissues and is rarely expressed in any other cell types including hematopoietic cells, muscle cells, neurons, fibroblasts, adipocytes, or endothelial cells. While gal-7 expression in normal epithelial cells has been relatively well documented, we are just beginning to understand its role in cancers of epithelial origin. For example, in breast cancer we found that gal-7 was exclusively expressed in aggressive subtypes including HER2-positive and basal-like breast cancer [5]. We also found that gal-7 expression in cell lines that express low or undetectable levels of gal-7 resulted in increased metastasis to the lung and bone and larger osteolytic lesions [5]. This is in contrast to earlier reports showing that gal-7 is absent in some types of malignant cells and may sensitize cells to apoptosis induced by chemotherapeutic agents [6-9]. Thus, while prior studies cannot predict whether any changes in gal-7 expression will result in resistance to apoptosis in epithelial cancers, differential expression patterns in epithelial cancers may represent a helpful biomarker. Differential expression of galectins may facilitate differential diagnoses if it is correlated with stage/grade, especially in cases where a morphological diagnosis is controversial. Accordingly, because galectins are implicated in a wide range of diseases, intensive efforts have been employed to identify highly selective and potent galectin inhibitors. In the present work, we investigated expression of gal-7 in EOC cells and studied its functional relevance.

## RESULTS

### *Gal-7* expression is increased in ovarian cancer tumors

**Figure 1:**
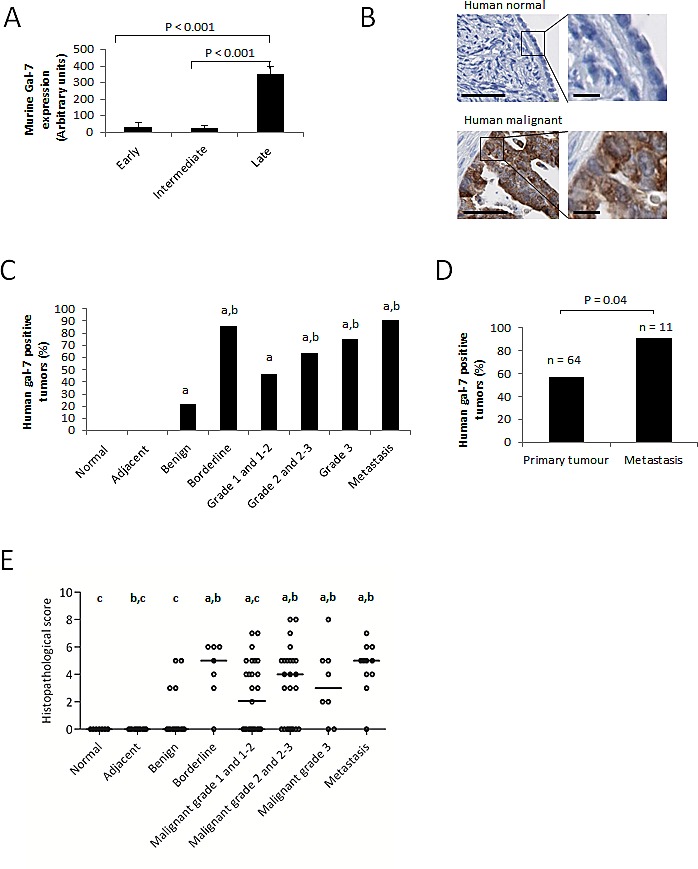
Expression of gal-7 in ovarian tissues (A) mRNA expression of *gal-7* in EOC at different stages of transformation. The data were obtained from a publicly accessible gene profiling of an *in vitro* mouse model of EOC progression [10]. (B) Representative IHC staining of gal-7 expression in normal or malignant human ovarian tissue samples. Bars represent 50 μm (left panel) and 10 μm (right panel) (C) Percentage of gal-7 positive samples in different subtypes of human ovarian cancer. (D) Comparative analysis between the percentages of human non-metastatic primary tumors and metastatic samples expressing gal-7. (E) Differences in IHC scorings of gal-7 expression among different human ovarian cancer subtypes. Error bars represent SEM. a: p ≤ 0.05 compared to normal tissue samples; b: p ≤ 0.05 compared to benign tumors samples; c: p ≤ 0.05 compared to metastatic samples.

**Figure 2:**
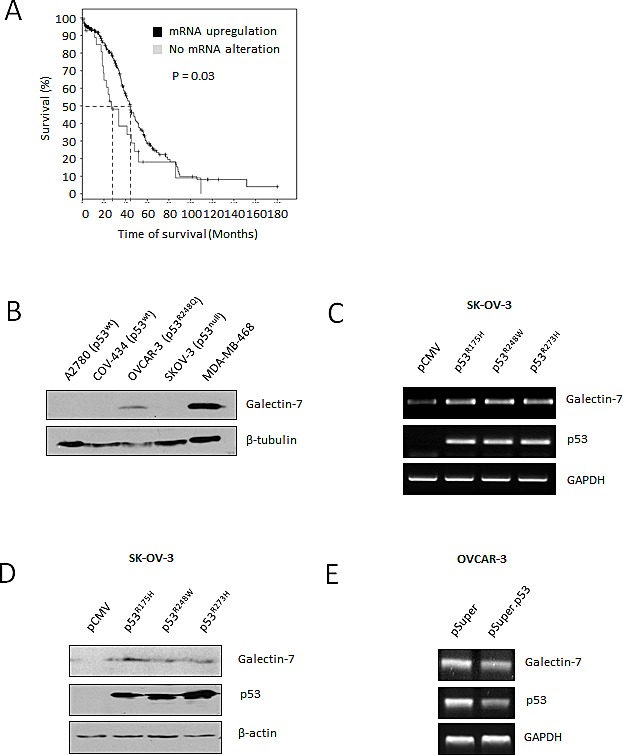
Gal-7 expression in EOC cells is associated to poor overall survival and is controlled by mutant forms of p53 (A) *In silico* analysis showing overall survival of ovarian serous cystadenocarcinoma cancer patients according to the mRNA expression level of *gal-7*. Data were obtained from the cBio portal RNAseq datasets [40]. (B) Western blot analysis of gal-7 expression in commonly used human ovarian cancer cell lines. The human MDA-MB-468 basal-like breast cancer cells, which readily express gal-7, were used as a positive control [12]. β-tubulin was used as an internal loading control. (C and D) *De novo* expression of *gal-7* as measured by RT-PCR and western blot respectively in SK-OV-3 transfected with expression vectors encoding mutant forms of human *p53*. An empty pCMV vector was used as a control for transfection. β-actin and GAPDH were used as internal loading controls. (E) RT-PCR analysis of *gal-7* mRNA expression in OVCAR-3 cells following suppression of endogenous p53 mutant. GAPDH was used as an internal loading control. Results are representative of three independent experiments.

To study the role of gal-7 in ovarian cancer, we first conducted an *in silico* analysis of *gal-7* gene expression in EOC cells using publically available datasets from the Gene Expression Omnibus (GEO) repository of the National Center for Biotechnology Information (NCBI). Interestingly, a microarray dataset (GDS1375) obtained from profiling a murine model of ovarian cancer indicated that while *gal-7* mRNA was rarely detectable at early and intermediate stages of transformation, it was expressed at significantly higher levels at later stages of transformation (P<0.001) (Fig. 1A) [10]. These data prompted us to assess gal-7 expression in human ovarian tissues by immunohistochemistry (IHC) using TMAs constructed from samples obtained from a cohort of 112 patients with different EOC histological subtypes and containing core samples from matched normal adjacent tissues (n=17) and normal (n=7) ovarian tissues. While we found no detectable expression of gal-7 in normal ovarian tissues, including the single layer of ovarian surface epithelium, gal-7 positive staining was detected in epithelial cells in all EOC histological subtypes (Table I and Fig. 1B-C). Positive staining for gal-7 was seen primarily in the cytoplasm of tumor cells (Fig. 1B). No correlation was found between expression of gal-7, the age of the patients or the stage of the disease (Supplementary Table S1). However, we found that gal-7 was present more frequently in metastatic samples compared to non-metastatic samples (P<0.05) (Fig. 1D). Histopathological scoring further revealed that gal-7 expression was significantly higher in high grade, borderline and metastatic tumors when compared to benign tumors. Low grade tumors also had significantly lower scores than metastatic samples (Fig. 1C and E). Analysis of the public RNAseq datasets obtained from the cBio Cancer Genomics Portal (http://cbioportal.org) also showed a significant correlation (P<0.03) between overexpression of *gal-7* mRNA and a lower overall survival of patients with ovarian serous cystadenocarcinoma (Fig. 2A and Supplementary Table S2).

**Table I:** Expression of gal-7 in ovarian tumors

	Gal-7 expression		Total
	Positive (n=44)	Negative (n=68)	(n=112)
Normal	0 (0 %)	7 (100 %)	7
Adjacent	0 (0 %)	17 (100 %)	17
Benign serous	3 (50 %)	3 (50 %)	6
Benign mucinous	1 (8.33 %)	11 (91.77 %)	12
Bordeline serous	5 (83.33 %)	1 (16.67 %)	6
Bordeline mucinous	1 (100 %)	0 (0 %)	1
Serous	14 (43.73 %)	18 (56.25 %)	32
Mucinous	5 (71.43 %)	2 (28.57 %)	7
Endometrioid	8 (61.54 %)	5 (38.46 %)	13
Transitional cell	4 (80 %)	1 (20 %)	5
Clear cell	3 (75 %)	1 (25 %)	4
Granular	0 (0 %)	2 (100 %)	2

### Gal-7 expression in ovarian cancer cells is induced by mutant forms of p53

High expression of gal-7 in cancer cells is somewhat paradoxical because gal-7 has generally been considered to be a pro-apoptotic protein under the control of p53 [11]. However, in breast cancer cells gal-7 can be induced by mutant p53 to promote cancer progression. These data may explain why gal-7 is often constitutively expressed in cancer cells harboring mutant forms of p53 [12]. To determine whether a similar scenario exists in ovarian cancer, we tested whether mutant forms of p53 that are commonly expressed in ovarian cancer could regulate gal-7 expression. According to the International Agency for Research on Cancer (IARC) database, mutations at codons His175, Met243 and Arg273 in the *p53* gene are the most frequently occurring mutations in ovarian cancer (Supplementary figure S1) [13]. Interestingly, OVCAR-3 cells, which harbor a p53^R248Q^ mutation, readily express gal-7 (Fig. 2B). In contrast, gal-7 was not detected in ovarian cancer cells harboring a wild-type p53 (A2780 and COV434 cells) or cells with a p53^null^ genotype (e.g., SK-OV-3) [13] Transfection of SK-OV-3 cells with expression vectors encoding p53 with mutations at codons 175, 273, and 248 all induced *de novo* expression of gal-7 at both the mRNA and protein level compared to cells transfected with a control (empty) vector (Fig. 2 C-D). The use of siRNA specific for p53 further support the idea that gal-7 is regulated by p53 mutants (Fig. 2E).

### Where do we find gal-7 in ovarian cancer cells?

**Figure 3:**
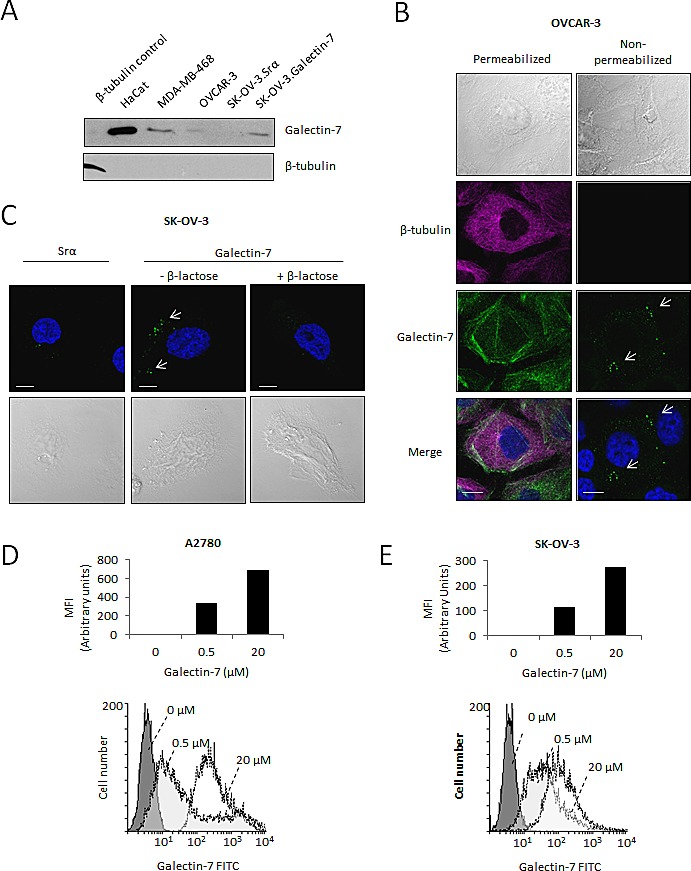
Release of gal-7 by ovarian cancer cell lines (A) Western blot analysis of cell culture supernatants collected from ovarian cancer cells. The HaCat keratinocyte cell line and the MDA-MB-468 cells were used as positive controls. (B) Confocal microscopy imaging of gal-7 (green) in ovcar-3 cells. Gal-7 was detected by immunofluorescence in permeabilized or non-permeabilized cells to detect the intracellular and extracellular protein, respectively. Staining of β-tubulin (magenta) was used as a control to monitor permeabilization. (C) Binding of extracellular gal-7 to SK-OV-3 cells transfected with the empty Srα (control) vector or the same vector encoding the *gal-7* gene. Cells were incubated with or without 0.1M β-lactose to inhibit the gal-7 binding to glycosylated receptors. Nuclei were stained with DAPI (blue). Bar represents 10 μm. (D) Flow cytometry analysis showing binding of gal-7 to the surface of A2780 and (E) SK-OV-3 cells. Binding assays were conducted using the indicated concentrations of FITC-labeled recombinant gal-7. MFI; mean fluorescent intensities. Results are representative of three independent experiments.

Our IHC staining in ovarian cancer tissues revealed that gal-7 was found mostly in the cytoplasm of cancerous cells. However, we found gal-7 in the supernatants of both OVCAR-3, and SK-OV-3 cells transfected with an expression vector encoding human gal-7 (Fig. 3A). The presence of gal-7 in the supernatants was confirmed by ELISA (with a range between 100 pcg/ml and 2.5 ng/ml). Confocal microscopy of permeabilized and non-permeabilized OVCAR-3 cells confirmed that endogenous gal-7 was also bound to the cell surface and in the cytoplasm (Fig. 3B). Similar data were obtained using SK-OV-3 cells transfected with an expression vector encoding gal-7 (Fig. 3C). Addition of β-lactose to the culture media decreased binding of gal-7 to SK-OV-3 cells, indicating that the binding was CRD-dependent. The ability of gal-7 to bind cell surface receptors of A2780 and SK-OV-3 cells was further confirmed by measuring the binding of FITC-labeled recombinant gal-7 by flow cytometry (Fig. 3 D-E). This binding was inhibited by the addition of β-lactose (Supplementary figure S2). Taken together, these results indicate that gal-7 exhibits dual localization in ovarian cancer cells, being found in both the extracellular compartment (cell surface and extracellular medium) as well as the intracellular compartment.

### Gal-7 increases the invasive behavior of ovarian cancer cells

**Figure 4:**
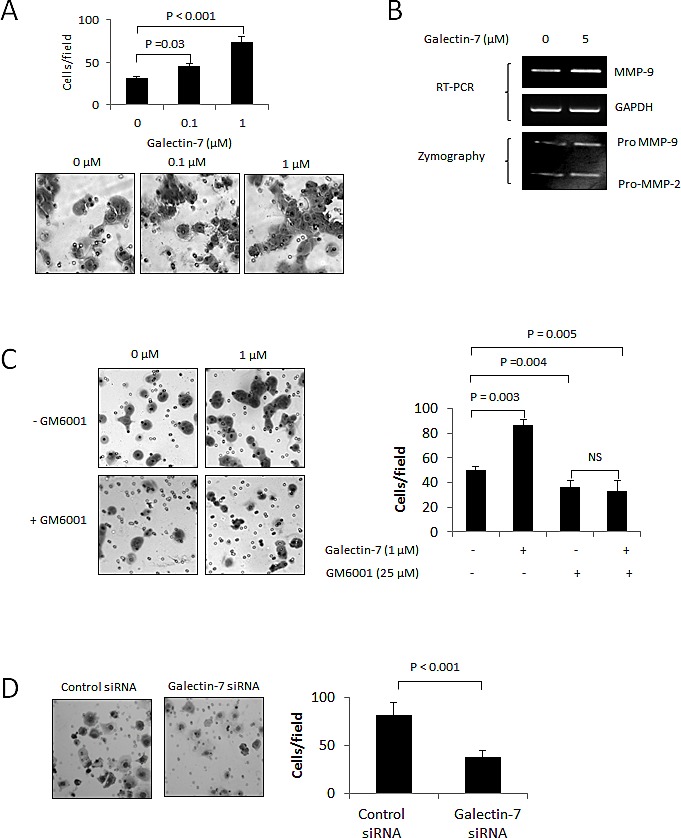

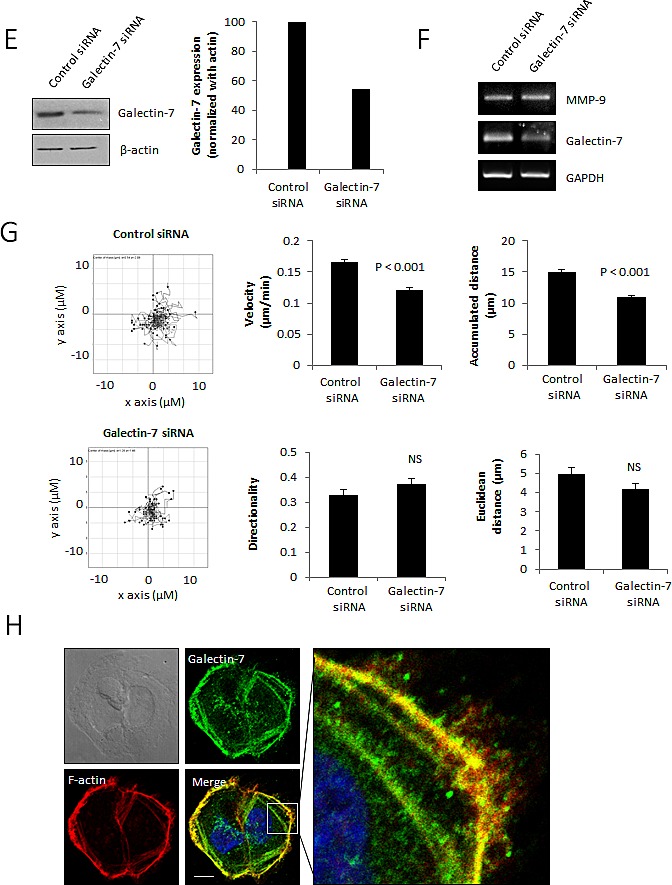
Gal-7 increases the invasive behavior of ovarian cancer cells (A) Migration of A2780 cells through Matrigel in absence and presence of increasing concentrations of recombinant gal-7. (B) RT-PCR and zymography showing expression of MMP-9 by A2780 cells with or without 5 μM recombinant gal-7. (C) Effect of GM6001 on gal-7-induced Matrigel invasion of A2780 cells. (D) Migration of OVCAR-3 cells through Matrigel following suppression of gal-7. (E) Western blot analysis showing specific gal-7 suppression by the siRNA. (F) Effect of gal-7 suppression on *MMP-9* mRNA expression and (G) *in vitro* cell motility. Velocity, accumulated distance, Euclidean distance and directionality were measured on individual OVCAR-3 cells (n = 60) tracked by live cell imaging using a scratch wound healing test. Images were captured every 10 min for 2 h. Error bars represent SEM. (H) Intracellular distribution of gal-7 inside OVCAR-3 cells. Gal-7 (green) and F-actin (red) were detected by immunofluorescence and visualized by confocal imagery. Nuclei were stained with DAPI (blue). Bar represents 10 μm. Results are representative of three independent experiments. Error bars represent SD except for (G) which are SEM.

Release of galectins in the tumor microenvironment has been shown to play a central role in cancer progression, most notably by increasing the invasive properties of cancer cells through lectin-dependent interactions (As Reviewed in [14]). To test whether this was the case for gal-7 in ovarian cancer cells, A2780 cells were treated with increasing concentrations of recombinant human gal-7 and tested for their invasive properties using a standard Matrigel invasion assay. We found that addition of gal-7 to A2780 cells induced a dose-dependent and significant increase in their invasive behavior (P<0.001) (Fig. 4A). Interestingly, this increase correlated with higher expression of MMP-9 (Fig. 4B). The use of GM6001, a broad range MMP inhibitor, significantly reduced the invasive behavior that was observed after treatment with recombinant gal-7 (P<0.005) (Fig. 4C). Inhibition of endogenous gal-7 expression by siRNA also induced a significant reduction in their invasive properties (P<0.001) (Fig. 4D-E). However, suppression of gal-7 did not correlate with reduced levels of MMP-9 (Fig. 4F), suggesting that gal-7 may also function independently of MMPs, or that other MMPs are involved.

Because gal-7 has been associated with the motility of mouse keratinocytes [15], we next investigated the effect of gal-7 on cell motility, using live cell video microscopy. We found that repression of gal-7 significantly reduced the velocity (P<0.001) and the accumulated migration distance (P<0.001) of OVCAR-3 cells without affecting the directionality or the Euclidean distance of migration (Fig. 4G). Interestingly, addition of recombinant gal-7 to A2780 cells had no effect on cellular motility, suggesting that intracellular rather than extracellular gal-7 is involved (Supplementary fig. S3). This possibility is real because gal-7 has previously been shown to co-localize with actin-rich structures (podosomes) implicated in cell motility [15]. Indeed, we found that gal-7 co-localized with cortical actin (Fig. 4H). Taken together, these results indicate that both intracellular and extracellular gal-7 increase the invasive behavior of ovarian cancer cells.

### Extracellular gal-7 induces apoptosis of lymphocytes and monocytes

**Figure 5:**
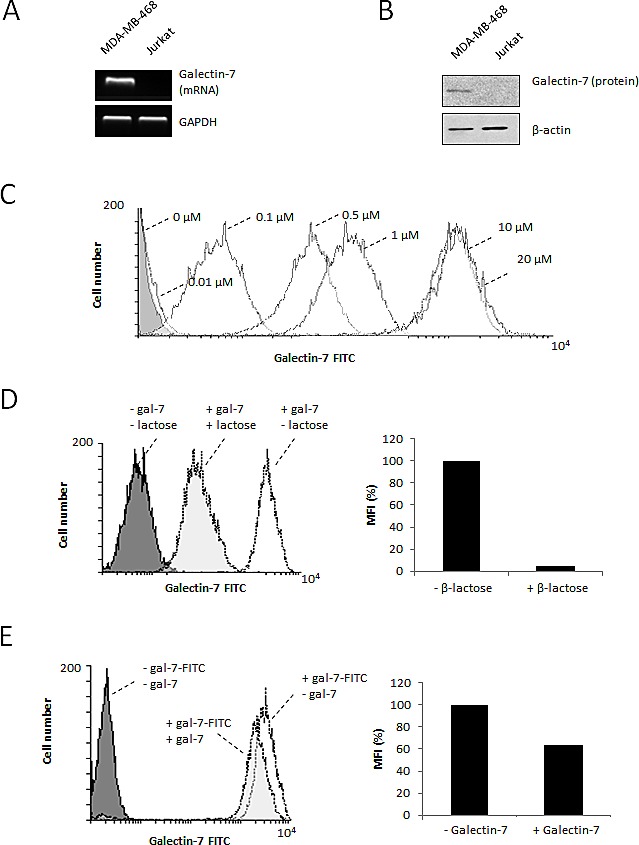
Binding of recombinant gal-7 to Jurkat cells RT-PCR (A) and western blot (B) analysis of gal-7 expression in Jurkat cells. (C) Binding of gal-7 to the surface of Jurkat T cells. Increasing concentrations of recombinant FITC-labeled gal-7 were added to Jurkat T cells. Binding was measured by flow cytometry after 30 min of incubation. In some experiments, binding was measured in presence or absence of (D) 0.1 M β-lactose or (E) 20 μM of non-conjugated (cold) recombinant gal-7. Results are representative of three independent experiments.

**Figure 6:**
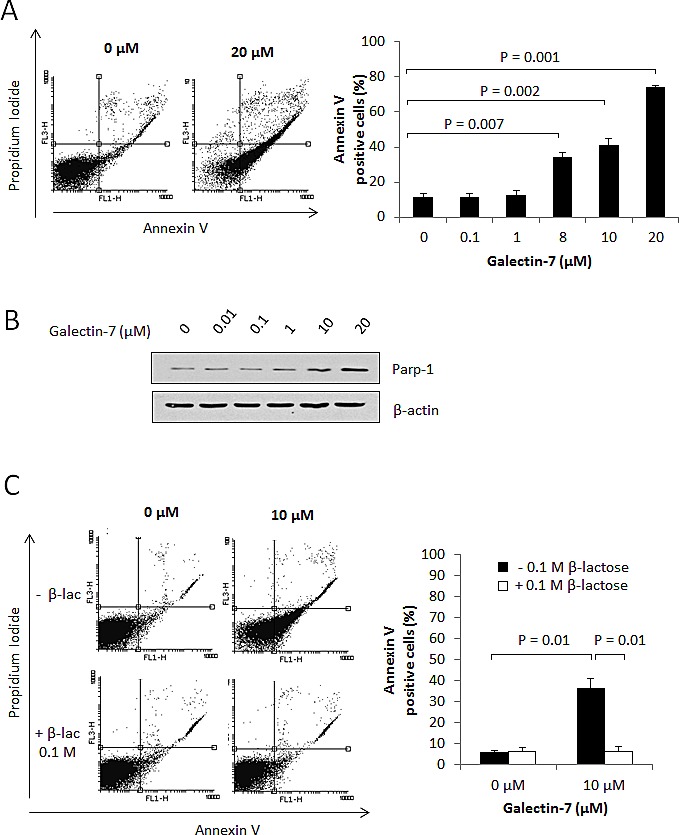

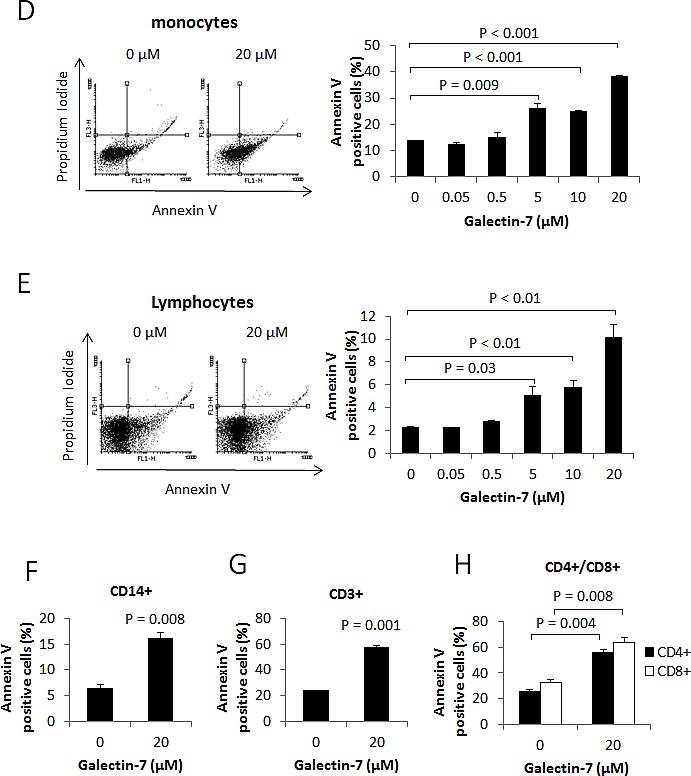
Gal-7 induces apoptosis of T cells Jurkat cells were treated for 4 h with increasing concentrations of recombinant gal-7. Apoptosis was monitored by measuring (A) Annexin V staining and (B) Parp-1 cleavage. (C) Effect of β-lactose on gal-7 induced apoptosis. (D-E) Freshly isolated PBMCs were collected from healthy individuals and treated for 4 h with increasing concentration of recombinant gal-7. Apoptosis was measured on gated populations of monocytes and lymphocytes and on cell populations expressing cell surface receptors (F-H). Cells in the lower right quadrant are representative of annexin-positive, early apoptotic cells. The cells in the upper right quadrant indicate Annexin-positive/PI-positive, late apoptotic cells. The percentage of cell death was obtained by adding the percentage of cells found in the lower and the upper right quadrants. Results are representative of three independent experiments. Error bars represent SD.

Galectins are well known for their ability to induce local immunosuppression within the tumor microenvironment. While this is well-known for galectin-1 and galectin-3, a recent report by Yamaguchi *et al.* suggests that this may well be the case for gal-7 [16]. We tested this possibility using Jurkat T cells, which do not express gal-7 mRNA or protein (Fig. 5A-B). Using FITC-labeled recombinant gal-7, we found that gal-7 bound to the surface of Jurkat T cells in a dose-dependent manner (Fig. 5C). This binding was inhibited by the addition of β-lactose and unlabeled recombinant gal-7, indicating that binding of gal-7 to Jurkat cells was dependent on its specific interaction with cell surface glycans (Fig. 5D-E). Flow cytometric analysis also revealed that binding of gal-7 to Jurkat T cells induced a significant increase in annexin V staining (Fig. 6A), suggesting that gal-7 triggers apoptosis of T cells. This hypothesis was supported by western blot analysis of PARP-1 cleavage (Fig. 6B). The effect of gal-7 on apoptosis was dependent on its lectin activity, as tested in experiments using lactose as an inhibiting sugar (Fig. 6C). Similar results were obtained using freshly isolated human PBMCs. Multiparametric analysis by flow cytometry showed that CD14-positive monocytes and CD4- or CD8-positive T cells all showed increased annexin V staining following incubation with recombinant gal-7 (Fig. 6D-H). Interestingly, although we found that gal-7 binds to the surface of ovarian cancer cells, it did not induce detectable apoptosis (Supplementary figure S4).

## DISCUSSION

There is a critical need to develop effective new strategies for the treatment of patients with EOC. In the present work, we examined the clinical significance of gal-7 overexpression in ovarian cancer and investigated its potential as a therapeutic target for the treatment of ovarian cancer. More specifically, we found that while gal-7 is not detected in normal ovarian tissues, its expression is induced in benign, borderline and malignant tumors. In fact, analysis of histopathological scores indicated that its expression is significantly augmented in high grade, borderline and metastatic tumors compared to benign tumors. Moreover, overexpression of *gal-7* mRNA correlated with a poor overall survival of patients with serous cystadenocarcinomas, which account for the vast majority of EOC. We found that mutant forms of p53 most likely induce gal-7 expression in ovarian cancer cells, because *de novo* expression of the most common mutant forms of p53 induced gal-7, while suppression of p53 by siRNA reduces gal-7 expression. Finally, our *in vitro* data support the idea that gal-7 expression has the potential to impact ovarian cancer progression by increasing the invasive properties of ovarian cancer cells and by killing immune cells. Taken together, our study validates the clinical significance of gal-7 overexpression in ovarian cancer and provides a rationale for targeting gal-7 to improve the outcome of patients with this disease.

Mutations in the *p53* gene are among the most common genetic alterations found in cancer. In ovarian cancer, these mutations are observed in more than 90% of the cases and are more frequently observed in later stage tumors compared to early stage tumors [17-20]. In many cases, the presence of specific mutations is associated with a gain of function, often by inducing expression of genes involved in various aspects of cancer biology [21]. However, the mechanism by which specific mutant forms of p53 can induce gal-7 in ovarian cancer cells is currently unknown. Our findings suggest that gal-7 might in fact be part of a gain-of-function pathway that is frequently observed in many types of cancer [21]. This possibility is supported by our data showing that gal-7 may promote cancer progression by increasing the invasive properties of ovarian cancer cells. While we found that recombinant gal-7 had no effect on cell motility, we found that repression of gal-7 decreased the velocity and accumulated distance of migration of OVCAR-3 cells. These data suggest that ovarian cancer cell motility was modulated by intracellular gal-7, which co-localized with the actin cytoskeleton within cortical actin. This finding is reminiscent of a previous report by Gendronneau *et al.* [15], who showed that keratinocytes isolated from gal-7 KO mice had reduced motility when compared to normal keratinocytes. They had also found that gal-7 was localized in the podosomes of these cells, indicating that gal-7 is involved in organization of the actin cytoskeleton. Such a role has been well documented for galectin-1, which binds to the actin cytoskeleton and participates in actin polymerization-depolymerization by modulating the activity of small GTPase [22, 23]. Alternatively, release of gal-7 in the tumor microenvironment by EOC cells may favor establishment of a local immunosuppressive response by killing immune cells. This possibility has been reported in the case of galectin-1, which is also overexpressed in EOC [24]. Galectin-1 is indeed released by SK-OV-3, but not OVCAR-3 cells [25]. Galectin-3 is also expressed in EOC, although it is mainly found inside the cells and does not vary among benign, borderline and malignant mucinous and serous tumors [26]. In fact, it is becoming clear that many cell types do express more than one intracellular galectin, suggesting existence of functional redundancy. For example, recombinant galectin-1, -3, -8 and -9 have all been shown to induce apoptosis in Jurkat T cells [16, 27-30]. However, in contrast to galectin-3, we found that killing of Jurkat T cells by gal-7 is independent of CD45 (Supplementary Fig. S5). This suggests that expression of galectins by EOC may induce apoptosis of specific infiltrating immune cell populations depending on their fine carbohydrate specificities [8, 31]. Such redundancy is often necessary to increase the maintenance of important gene functions and to limit losses following mutations/deletions of specific genes (functional compensation). Future *in vivo* investigations are required to determine how the “galectinome” of EOC carves development of local immune suppression and whether development of CRD-specific galectin inhibitors is an attractive avenue for the treatment of EOC.

Although relatively high concentrations of soluble gal-7 were required to trigger apoptosis or cell invasion *in vitro*, the concentrations we used for our *in vitro* studies, however, were comparable to concentrations commonly used to study galectin-apoptosis in T cells *in vitro*. Such range of concentrations are not unexpected since *in vitro* studies are usually carried out using recombinant galectins that display suboptimal biological activity [32]. It is also important to note that extracellular galectins bind to a relatively large number of cell surface glycoprotein receptors [33], which is known to concentrate galectins by forming spatial gradients that allow them to exert their biological activity following binding to their respective receptors localized on responding cells [34, 35]. In fact, total galectin concentrations in tissues can reach up to 70-80 mg/kg tissue or approximately 1-2 μM [36]. Thus, miroenvironmental concentrations of gal-7 in ovarian cancer could be quite high, and may explain, at least in part, why galectins are now considered as key mediators of development of distant metastasis through induction of local and systemic immunosuppression [37-39]. It will be interesting to determine whether gal-7 is found in ascites of patients with ovarian cancer and whether levels of gal-7 correlate with disease progression, remission and/or response to treatment.

It is becoming clear that gal-7 plays a central role in cancer of epithelial origin. There are now numerous reports showing that gal-7 can modulate the progression of many types of carcinoma. This has been well established in breast cancer cells. In fact, there is increasing evidence that targeting galectins in cancer may help to reduce immunosuppression during metastatic progression of the disease [38]. In EOC, our findings add to a recent report by Kim *et al.* who showed that gal-7 was associated with a lower survival rate of Korean patients with EOC [26]. While the authors reported that gal-7 expression in tissue samples was primarily detected in the nuclei and occasionally in the cytoplasm, our IHC, confocal microscopy and western blot analysis suggest that extracellular gal-7 is released outside the cells and may have a significant impact on tumor progression by inducing immunosuppression and increasing the invasive behavior of tumor cells. Thus, these functions may explain Kim's findings and ours showing that gal-7 is associated with metastasis and poor overall survival. Taken together, these studies provide proof-of-principle that targeting gal-7 may represent a valuable strategy to overcome cancer-associated immunosuppression and dissemination of metastasis in EOC, restrain tumor growth and prevent metastatic disease.

## MATERIAL AND METHODS

### Cell lines and reagents

The COV-434, HACAT and MDA-MB-468 cell lines were maintained in Dulbecco's modified Eagle medium (DMEM). The A2780, Jurkat and OVCAR-3 cells were maintained in RPMI 1640 medium, and the SK-OV-3 cell line was maintained in McCoy's 5A medium. The culture medium were supplemented with 10% [v/v] fetal bovine serum, 2 mmol/L l-glutamine, 10 mmol/L HEPES buffer, and 1 mmol/L sodium pyruvate. All cell culture products were purchased from Life Technologies (Burlington, ON, Canada).

### Vectors and transfections

The vectors encoding mutant p53 (R175H, plasmid 16436; R273H, plasmid 16439; R248W, plasmid 16437) were obtained from Addgene (Cambridge, MA, USA). pSuper and pSuper-p53 siRNA vectors were kindly provided by Dr. Reuven Agami (The Netherlands Cancer Institute, Amsterdam, Netherlands). The empty pCMV vector was produced by excising the p53 cDNA from the p53^R175H^ vector using the EcoRI restriction enzyme. Control siRNA and gal-7 siRNA were purchased from Santa Cruz (Dallas, Texas, USA). cDNA encoding the human *gal-7* gene has been previously described [39]. For transfection, the cells were plated at equal densities 24 h prior to transfection and then transfected with the indicated vectors or siRNAs using the Lipofectamine 2000 reagent (Life Technologies) according to the manufacturer's protocol.

### Immunohistochemistry

Ovarian tissue microarrays (TMA) were purchased from Lifespan Biosciences (Seattle, WA, USA) and US Biomax (Rockville, MD, USA). Immunostaining reactions for gal-7 were performed using the Discovery XT automated immunostainer (Ventana Medical Systems, Tucson, AZ, USA). Deparaffinized sections were incubated in cell conditioning 1 (pH 8.0) buffer for antigen retrieval and then stained for 60 min with the anti-human gal-7 polyclonal antibody (R&D Systems, Minneapolis, MN) using a 1:150 dilution. The slides were counterstained with hematoxylin and scanned at a high resolution using the Nanozoomer Digital Pathology (Hamamatsu, Bridgewater, NJ). The percentage of staining was scored from 0 to 4 according to the percentage of positive cells displaying gal-7 expression within a sample (0 = 0%; 1 ≤ 25%; 2 ≤ 50%; 3 ≤ 75%; 4 ≤ 100%). The intensity of staining was also scored from 0 to 4, with a score of 0 representing no staining and a score of 4 representing the strongest staining observed. Histological scores were calculated by adding both scores.

### Immunofluorescence

Cells were fixed in paraformaldehyde 3% [w/v] for 15 min, permeabilized in PBS/Triton X-100 0.1% [v/v] for 5 min and blocked 30 min at 4°C in 1% [w/v] PBS/BSA. Goat anti-human gal-7 (dilution 1:100), rabbit anti-β-tubulin (dilution 1:100, New England Biolabs), rabbit anti-goat Alexa Fluor 488 (dilution 1:500, Life Technologies) and donkey anti-rabbit Alexa Fluor 647 (dilution 1:500, Life Technologies) antibodies were used. Filamentous actin was stained with Alexa Fluor 594 conjugated-phalloidin (dilution 1:500, Life Technologies). All antisera were diluted in PBA and all washing steps were performed with PBS. Nuclei were stained with ProLong Gold Antifade Reagent with DAPI (Life Technologies). Cells were visualized with a LSM 780 laser-scanning microscope (Zeiss, Jena, Germany).

### RNA isolation and RT-PCR

Total cellular RNA was isolated from cells using the TRIzol reagent (Life Technologies) according to the manufacturer's instructions. First-strand cDNA was prepared from 2 μg of cellular RNA in a total reaction volume of 20 μL using the reverse transcriptase Omniscript (QIAGEN, Mississauga, ON, Canada). After reverse transcription, the human *gal-7* (gene ID 3963, forward primer: 5’- TCC CAA TGC CAG CAG GTT CCA TGT -3’ and reverse primer: 5’- GAA GCC GTC GTC TGA CGC GAT GAT-3’), *TP53* (gene ID 3963, forward primer: 5’- CCAGCCAAAGAAGAAACCAC-3’ and reverse primer 5’- TATGGCGGGAGGTAGACTGA-3’), *MMP-9* (gene ID 4318, forward primer 5’- caacatcacctattggatcc-3’ and reverse primer 5’ cgggtgtagagtctctcgct-3’) and *GAPDH* (gene ID 2597, forward primer: 5’- CGG AGT CAA CGG ATT TGG TCG TAT-3’ and reverse primer: 5’-CAG AAG TGG TGG TAC CTC TTC CGA -3’) cDNAs were amplified using the following conditions: 94°C for 3 min, followed by 30 to 35 cycles of the following: 94°C for 40 sec, 60°C for 40 sec, and 72°C for 40 sec, followed by a final extension step at 72°C for 10 min. PCR was performed in a thermal cycler (MJ Research, Watertown, MA). The amplified products were analyzed by electrophoresis using 1.5% [w/v] agarose gels, SYBR Safe (Life Technologies) DNA gel staining and UV illumination.

### Western blotting and ELISA analysis

For whole cell extracts, cells were homogenized and resuspended in RIPA buffer (Thermo Fisher Scientific, Rockford, IL, USA) containing protease inhibitors (Roche, Laval, QC, Canada). For extracellular media, cells were cultured to confluence in 12-wells plates in 600 μl of media for 48 h. Media was centrifuged at 3,000 rpm for 10 min to remove cell debris. Equal amounts of whole-cells extract (20 to 60 μg) or extracellular media (40 μl) were separated on SDS-polyacrylamide gel and transferred onto nitrocellulose membranes (Bio-Rad Laboratories, Mississauga, ON, Canada). The membranes were first blocked with 5% milk [w/v] in TBS/0.5% Tween 20 [v/v] for 60 min at room temperature and subsequently blotted overnight in a solution of TBS containing 3% BSA [w/v] and 0.5% Tween 20 [v/v]. The following antibodies were used: a goat anti-gal-7 polyclonal antibody (1:1000), a rabbit anti-Poly-(ADP-ribose) polymerase (PARP)-1 (p25) polyclonal antibody (1:5000; Epitomics, Burlingame, CA, USA), a rabbit anti-p53 (1:1000; Santa Cruz), a mouse anti-β-actin (1:20000; Sigma-Aldrich, St. Louis, MO, USA) and a rabbit anti-β-tubulin (1:1000) polyclonal antibodies. Secondary antibodies consisted of horseradish peroxidase-conjugated donkey anti-rabbit (GE Healthcare, Buckinghamshire, England), donkey anti-goat (R&D Systems) or sheep anti-mouse (GE Healthcare) IgG. Detection was performed using the enhanced chemiluminescence method (GE Healthcare). For extracellular media, an ELISA was performed according to the manufacturer's recommendations (Ray Biotech, Norcross, GA, USA).

### Gelatin zymography

A confluent monolayer of cells was seeded on a 24-well plate in 300 μl serum-free RPMI medium. The supernatant was harvested after 24 hr and centrifuged at 3,000 rpm to remove cell debris. To increase the protein concentration, 200 μl of media was concentrated by centrifugation for 1 h at 60°C in a SpeedVac system. A total of 40 μl was then electrophoresed under non-reducing conditions using 7.5% SDS-polyacrylamide gels containing 1 mg/ml gelatin (Merck, Darmstadt, Germany). To remove SDS after the electrophoresis, the gel was washed twice for 1 h in a solution containing 50 mM Tris, 5 nM CaCl2, 0.2% [v/v] NaN_3_ and 2.5% [v/v] Triton X-100. After an overnight incubation in the same buffer containing only 1% [v/v] Triton X-100, the gels were stained with Coomassie brilliant blue and destained in 45% methanol 15 % acetic acid [v/v]. Gelatinolytic activity was identified as a clear band on a blue background. The lowest band (72 kDa) corresponds to MMP-2, the highest band (92 kDa) to MMP-9.

### Production of recombinant gal-7

Gal-7 cDNA was cloned into pET-22b(+) using NdeI and HindIII restriction enzymes. The protein was produced in *E. coli* BL21 cells (DE3) at 37°C. Isopropyl β-D-1-thiogalactopyranoside (IPTG) (1 mM) was added to the bacteria culture at an OD_600nm_ = 0.6-0.7 and the bacteria were further incubated for 4 h. Bacterial pellets were resuspended in lysis buffer (0.7 mg/mL lysozyme, 10 mM Tris pH 8, 100 mM NaCl, 1 mM EDTA, 1 mM DTT and protease inhibitor cocktail), incubated for 1 h at 37°C and centrifuged for 30 min at 15,000 x *g* (4°C). The supernatant was then filtered and applied to a lactose-agarose column and the protein was eluted in 1 mL fractions with 150 mM lactose solution. Purified fractions were analyzed by SDS-PAGE. Gal-7 was dialyzed against 20 mM potassium phosphate at pH 7.2 for all subsequent experiments.

### FITC conjugation and gal-7 binding assay

Briefly, 10 μl of a 2 mg/ml fluorescein isothiocyanate (FITC)/DMSO solution was added to 300 μl of 1.7 μg/μl recombinant gal-7 in a 0.1 M NaHCO_3_ pH 9.2 solution and incubated for 2 h at room temperature on a roller. FITC-conjugated gal-7 was then purified using a PD-10 Sepharose column (GE healthcare) and eluted with PBA containing 0.01% [v/v] sodium azide. To measure FITC-gal-7 binding to the cell surface, 2.5 × 10^5^ cells were incubated for 30 min with the indicated concentrations of gal-7 and then washed twice with PBA and resuspended in 500 μl PBA. For the competition assays, 0.1 M β-lactose or 20 μM non-conjugated gal-7 were added to the cells at 4°C for 30 min prior to the addition of FITC-conjugated gal-7. The samples were analyzed using a FACSCalibur (BD Biosciences) and the Flowing Software.

### Annexin V/PI staining

Apoptosis was measured by flow cytometry using Annexin V conjugated with Alexa Fluor 488 (Life Technologies) and propidium iodide. Briefly, 2.5 × 10^5^ cells were incubated with different concentrations of recombinant gal-7 at 37°C for 4 h. The cells were washed once in PBS and once in binding buffer (0.01 M HEPES, 0.14 M NaCl, 2.5 mM CaCl_2_, pH 7.4). Subsequently, the cells were stained for 15 min with Annexin V (2.5 μl Annexin V in 50 μl binding buffer) in the dark at room temperature. A total of 400 μl of binding buffer containing 0.25 μg/ml propidium iodide was added to the cells before analysis by flow cytometry.

### PBMCs isolation and characterization

Monocytes and lymphocytes were isolated from 30 ml heparinized blood. The blood was diluted 1:1 with ice-cold 2% (v/v) FBS-PBS and layered onto a Lymphoprep density gradient solution (Stemcell Technologies, Vancouver, BC, Canada) before centrifugation at room temperature for 20 min at 800 x *g*. The PBMC interphase was washed twice in RPMI 1640 and resuspended in RPMI-1640 containing 10% (v/v) FBS. Immunophenotyping of monocytes and T cell subpopulations were performed by membrane immunofluorescence staining of cell surface markers using the following antibodies: mouse anti-human phycoerythrin (PE)-conjugated CD14, mouse anti-human allophycocyanin (APC)-conjugated CD3, mouse anti-human CD4 PE-conjugated, mouse anti-human APC-conjugated CD8. Each analysis was carried out with at least 10,000 cells. All antibodies were purchased from BD Biosciences.

### Invasion assay

Serum-induced cell invasion was examined using a 24-well Matrigel invasion chamber with 8-μm pores membrane. A total of 2 × 10^5^ cells were incubated in the upper chamber in serum-free medium. The lower chamber contained 10% [v/v] FBS. After 24 h, the upper surface of the insert was wiped gently with a cotton swab to remove non-migrating cells. Cells that had migrated to the lower surface of the membrane were stained with 1% borax/1% toluidine blue [w/v] and counted separately under a microscope. To inhibit endogenous extracellular proteases, the same volume of GM 6001 negative control solution (Santa Cruz) or GM 6001 (Santa Cruz) was directly added into the upper chamber and incubated with cells for the whole duration of the experiment.

### Live cell imaging

One day prior to the experiment, a confluent monolayer of cells was seeded into a 6-well glass bottom culture plate (MatTek Corporation, Ashland, MA, USA). A scratch with a pipette tip was made within the cell monolayer, followed by a wash with PBS to remove cell debris. The plate was moved to an incubator PM S1 and migration was visualized with a LSM 780 laser scanning microscope. Images were captured every 10 min for the indicated time. For each condition, the movement of 30 to 60 different cells was recorded. Cell movement was analyzed using the Image J plugins manual tracking and chemotaxis tool.

### Statistical analysis

Statistical significance of the experiments was evaluated using the unpaired Student's t-test or the Fisher's exact test. Results were considered statistically significant at P≤0.05.

## SUPPLEMENTARY MATERIAL FIGURES AND TABLES


