# Supplementary material for: Expression and functions of galectin-7 in ovarian cancer
Source: Oncotarget. 2014 Jul 31;5(17):7705–21. doi: 10.18632/oncotarget.2299 (PMC4202155; doi:10.18632/oncotarget.2299)
Supplement: Supplementary file 1 [file oncotarget-05-7705-s001.pdf]

Expression and functions of galectin-7 in ovarian cancer

Supplementary Material

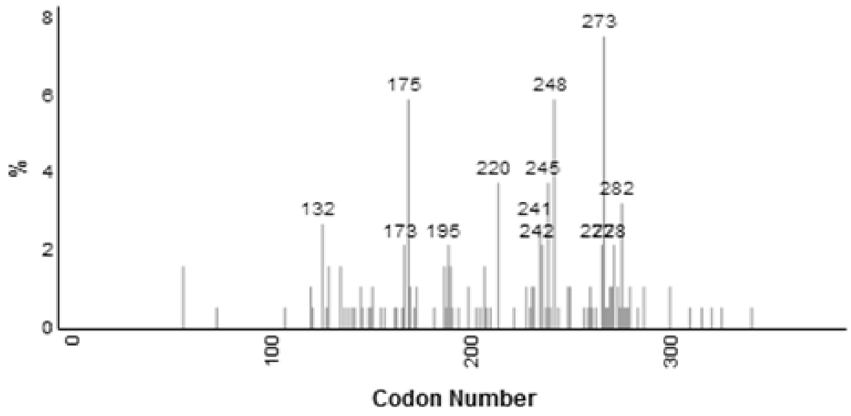

Supplementary figure S1: Must common p53 mutations in ovarian cancer cells. Frequency and codon distribution of p53 mutation among ovarian cancer cells, according to IARC database (n=186) [14].

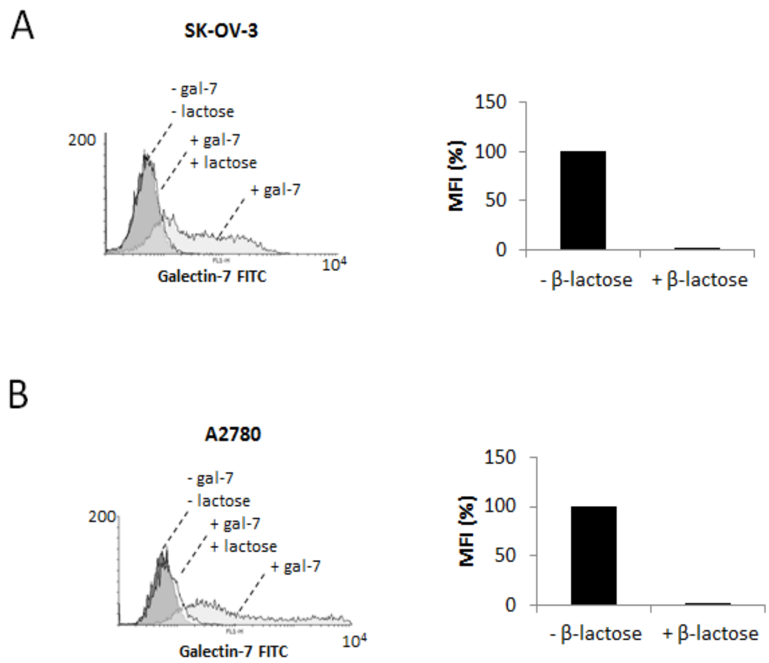

Supplementary figure S2: Binding of recombinant gal-7 on SK-OV-3 and A2780 cells. 0.5 µM recombinant FITC-labeled gal-7 was added to SK-OV-3 and A2780 cells in the presence or absence of 0.1 M β-lactose. Binding was measured by flow cytometry. Results are representative of three independent experiments.

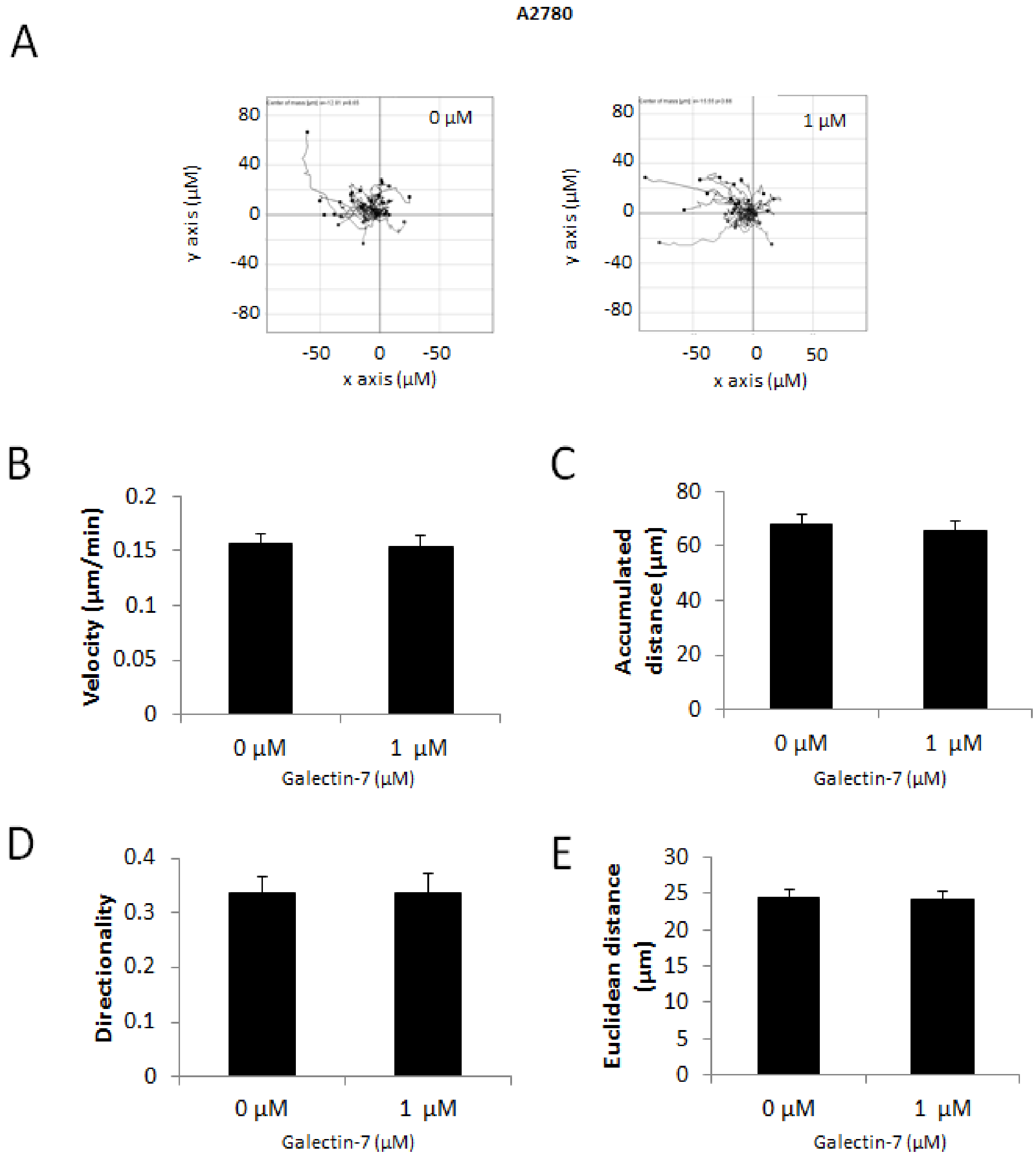

Supplementary figure S3: Recombinant galectin-7 does not modulate the motility of A2780 cells. A2780 cells were seeded into a 6-well glass bottom culture plate. Cells were treated with or without 1 µM recombinant galectin-7, 2 h prior to the experiment. A scratch was made and images were captured every 15 min for 7.5 h. **(A)** Plots represent 30 cells/sample, tracked by live cell imaging during scratch wound healing test. Histograms represent the **(B)** velocity, **(C)** accumulated distance, **(D)** Euclidean distance and **(E)** directionality of the cells. Error bars represent SEM. Results are representative of three independent experiments.

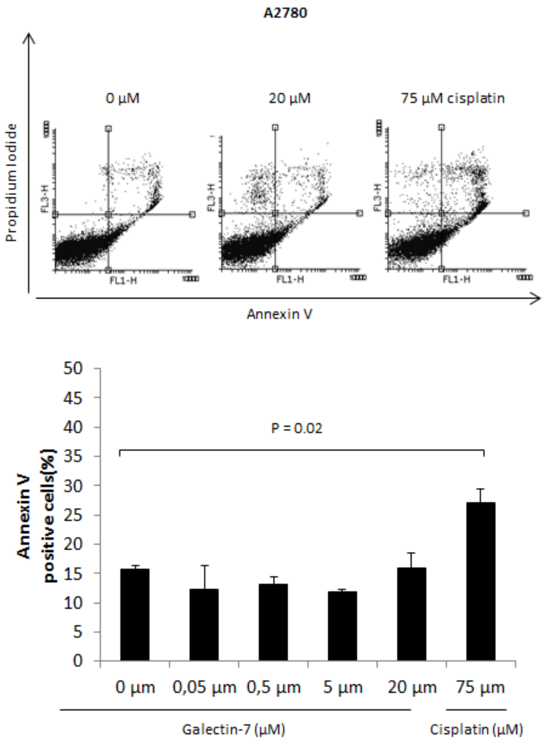

Supplementary figure S4: Extracellular galectin-7 does not induce apoptosis of A2780 cells. A2780 cells were treated for 4 h with increasing concentration of recombinant galectin-7. As a positive control of apoptosis, cells were also treated with 75 µM cisplatin. The induction of apoptosis was determined by flow cytometric analysis of Annexin V-alexa 488 and PI-staining. Cells in the lower right quadrant indicate Annexin-positive, early apoptotic cells. The cells in the upper right quadrant indicate Annexin-positive/PI-positive, late apoptotic cells. Results are representative of three independent experiments. Error bars represent SD.

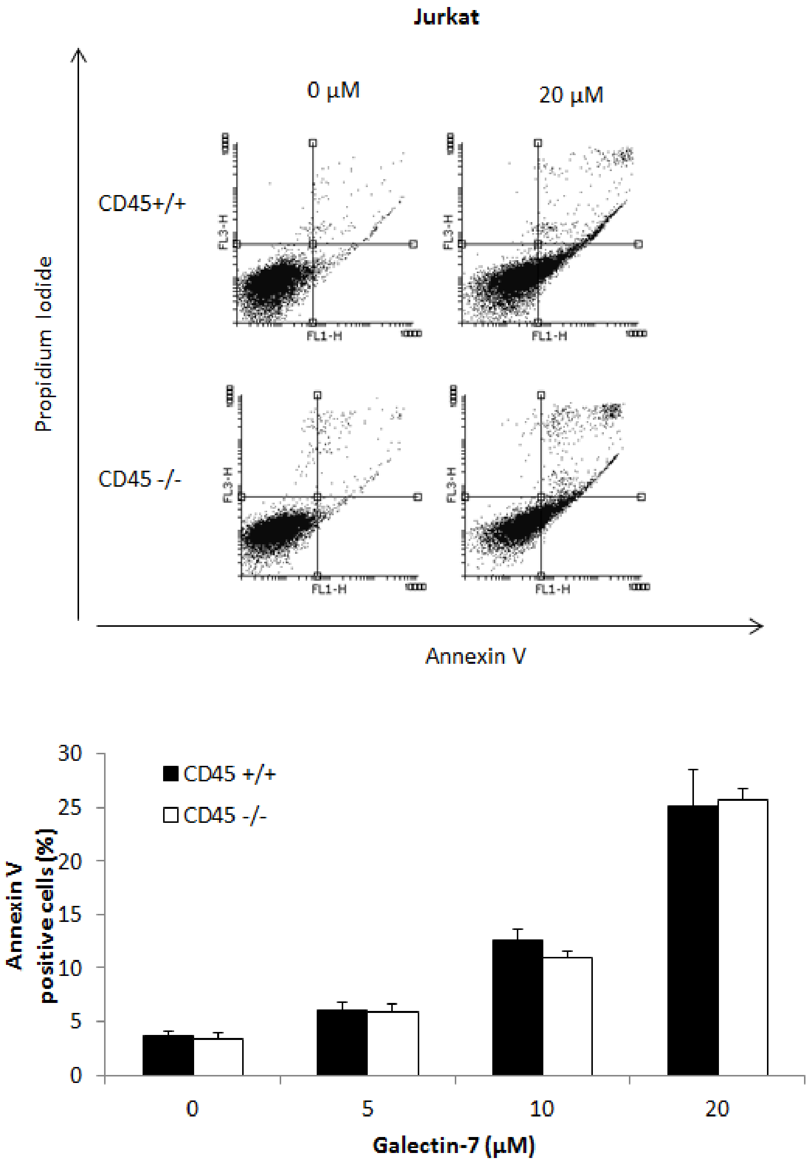

Supplementary figure S5: Extracellular galectin-7 induces apoptosis of Jurkat cells, independently of CD45 receptor. $CD45^{+/+}$ and $CD45^{-/-}$ Jurkat cells were treated for 4 h with or without 20 µM recombinant galectin-7. The induction of apoptosis was determined by flow cytometric analysis of Annexin V-alexa 488 and PI-staining. Cells in the lower right quadrant indicate Annexin-positive, early apoptotic cells. The cells in the upper right quadrant indicate Annexin-positive/PI-positive, late apoptotic cells. Results are representative of three independent experiments. Error bars represent SD.

Supplementary Table S1: Patients characteristics according to galectin-7 expression.

Outcome	All cases (n=64)	Galectin-7 expression Positive	Negative	P value
Median age, years (range)	48 (22-75)	50 (33-75)	45 (22-68)	0.15
Stage				0.99
I	43	25	18	
II	10	5	5	
III	11	6	5	
Grade				0.12
1 and 1-2	31	13	18	
2 and 2-3	25	16	9	
3	8	6	2	

Supplementary Table S2: Overall survival of ovarian serous cystadenocarcinoma cancer patients according to the mRNA expression level of gal-7.

	Total number of patients	Number of deceased patients	Median of survival (Months)
Overexpression	28	21	26.94
Unaltered expression	230	126	44.06

[a]Data were obtained from the cBio portal RNAseq datasets [10].
